# O‐TO‐T Advancement Reconstruction for Partial Glossectomy Defects: A Case Series

**DOI:** 10.1002/oto2.70015

**Published:** 2024-12-13

**Authors:** Kaersti L. Rickels, Aryan Shay, James R. Gardner, Deanne King, Jumin Sunde, Mauricio Moreno, Emre Vural

**Affiliations:** ^1^ Department of Otolaryngology–Head and Neck Surgery University of Arkansas for Medical Sciences Little Rock Arkansas USA

**Keywords:** floor of mouth, glossectomy, O‐to‐T, reconstruction, tongue

## Abstract

We present O‐T advancement reconstruction (OTAR) in lateral tongue defects, describing technique, indications, outcomes, and limitations. 11 patients with lateral tongue defects who underwent OTAR after earlystage cancer removal. Demographics, staging, functional oral intake scale (FOIS), dysphagia outcome severity scale (DOSS), defect size, and complications were included. Functional outcomes assessed through telephone encounters. Preoperative FOIS and DOSS were 6.9 and 6.8, postoperatively were 4.8 and 5. Mean defect of 4.7 cm × 3.4 cm. Nine patients required nasogastric tubes postoperatively. Site complications included 2 minor dehiscence. By telephone, tongue‐biting was reported in 3, mild dysarthria in 3, and food impaction in 2. FOIS and DOSS were 6.7 and 6.5. Reconstruction of tongue defects may be achieved with OTAR as a reliable alternative to primary closure or even more complex microvascular techniques. Utilization may preserve functional swallowing and speech outcomes, most probably due to lateral sulcus sparing features.

Tongue cancer remains the most common type of oral cancer^1^ and surgery is still the primary treatment of choice for this disease. When considering reconstruction, surgeons should emphasize not only on sound oncologic control but also the preservation of a given patient's health‐related quality of life (HRQoL).^2^, ^3^ With both goals in mind, the surgeon must be creative and critical when choosing which reconstructive technique might provide the best outcome. There are multiple techniques available for repair after partial glossectomies that depend on the location and extent of the tumor. When considering all reconstructive options, primary closure, whenever feasible, remains the easiest option for partial glossectomy defects. However, this technique may often cause limited mobility and deviation of the tongue towards the alveolus with an obliterated lateral sulcus between the tongue and the alveolus. With large wedge excisions, primary repair additionally may also lead to tip deviation, further impairing future tongue mobility. Various adaptations of primary closure techniques, utilizing tongue advancement or tissue rearrangement have been utilized in the past to overcome these concerns with primary closure.

We aim to present our lateral floor of mouth sparing O‐T advancement reconstruction (OTAR) for lateral partial oral tongue defects, which has not been characterized in the literature in detail to our knowledge.

## Methods

Upon approval by the institutional review board, we reviewed the medical records of 11 patients with lateral oral tongue defects related to the excision of early‐stage oral tongue cancers (T1 and T2) or pre‐cancerous lesions who underwent immediate local reconstruction using the OTAR technique. The majority of patients are part of the NRG‐HN006 Trial of Sentinel Lymph Node Biopsy Versus Elective Neck Dissection Trial Protocol, thus guiding management decisions. These reconstructions were all performed from May 2020 to February 2023 by 1 surgeon. We excluded patients with a previous history of head and neck radiation or prior glossectomy, with through‐and‐through defects in the neck, or those who were edentulous.

We collected basic demographic information including age, sex, BMI, smoking, and alcohol status. Additionally, we utilized multiple classification systems to demonstrate each patients' level of function and 10‐year survival with the Charlson Comorbidity Index (CCI), American Society of Anesthesiologists (ASA), and Eastern Cooperative Oncology Group (ECOG) scoring. We also collected clinical details including type of cancer, time of diagnosis, presenting symptoms, and prior radiation or chemotherapy exposure. Other preoperative instruments included clinical swallowing evaluation (CSE), Functional Oral Intake Scale (FOIS), and Dysphagia Outcome Severity Scale (DOSS) if available. In addition, the following surgical information was collected: preoperative imaging, tumor location and staging, defect size, pathology report and staging, and complications.

We conducted additional questions and assessments of postoperative functional outcomes through a single telephone encounter. The main instruments used to collect this data were the DOSS and the FOIS. In addition, presence and severity of dysarthria was investigated through subjective patient reporting and if present was classified as mild, moderate, or severe depending on patient's characterization of their perceived phonation. Patients were also asked about tongue biting and oral cavity food impaction.

**Figure 1:**
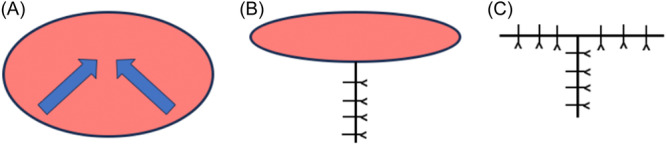
Simple demonstration of the O‐T advancement reconstruction technique showing that recruitment of tissue from both the partial floor of the mouth and oral tongue, as opposed to a single site, will limit tethering. Advancement of the anteroinferior and posteroinferior wound edge together to form the vertical limb (A). Closure of the vertical limb leading to a smaller superior defect site (B). Closure of the superior limb in a dorsal‐ventral fashion (C).

**Figure 2:**
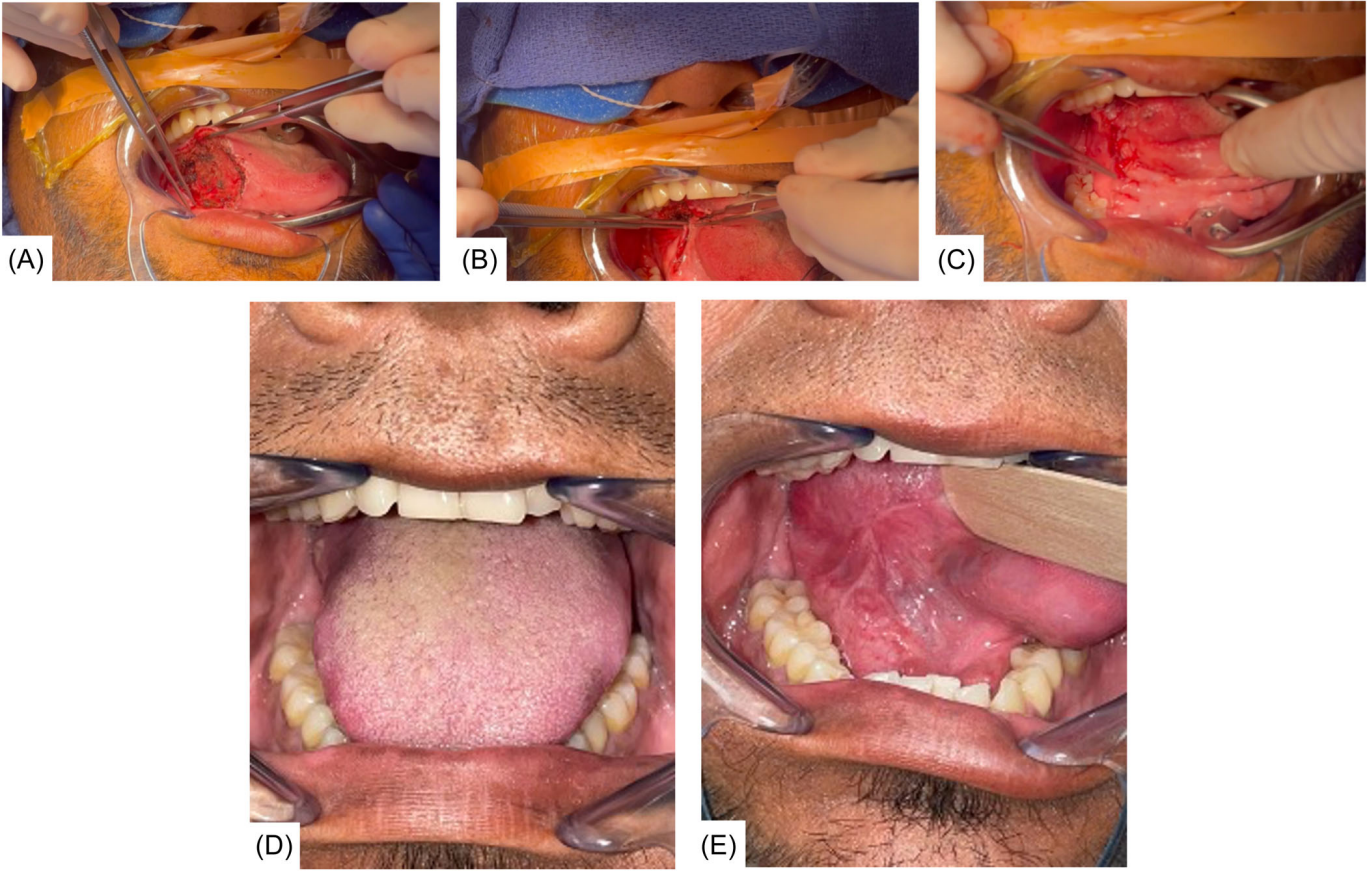
O‐T advancement reconstruction (OTAR) technique and postoperative appearance. (A) Example of right lateral tongue defect appropriate for OTAR (B) Tissue advancement for primary closure using OTAR. (C) Final closure of defect with OTAR. (D) Postoperative appearance at 3 months showing symmetric tongue and midline at rest. (E) Postoperative appearance at 3 months without tethering to the lateral sulcus.

Our surgical technique is as follows: After initiation of general anesthesia, patients underwent naso‐tracheal intubation. Exposure is achieved using a cheek retractor, side‐biter, and tongue stitch. Following oncologic excision with intraoperative frozen section margin analysis and neck dissection if indicated, the defect site is measured. The site is irrigated with saline and carefully inspected for hemostasis, using electrocautery as indicated. OTAR is deemed appropriate when the defect is laterally based, and when primary closure would lead to significant tethering of the tongue towards the lateral sulcus. Additionally, excision should not involve the floor of the mouth or the anterior tongue tip. Next, conservative undermining of the floor of mouth and tongue mucosae are performed. The wound edges are then approximated starting with the inferior limb, which can typically be closed in a single layer using 3‐0 Vicryl. Next, the superior limb is closed with 3‐0 Vicryl usually in a single layer. Tongue mobility is reassessed throughout the process by manual manipulation to ensure midline symmetry and appropriate protrusion. While this deviation was not explicitly measured intraoperatively, if the tongue deviated more than 1 cm from midline we deemed this technique inappropriate. A nasogastric feeding tube is then placed and secured with a bridle. Figure 1 illustrates this technique. Inoperative photos of the technique and the postoperative result may be seen in Figure 2.

We collected data with Research Electronic Data Capture (REDCap) and performed data analysis using descriptive statistics.

## Results

Demographic and patient characteristics can be found in Table 1. Of the 11 patients identified, the median age of the patients was 67 years (range 37‐92) and 8 were male (72.7%), 3 were female (27.3%). T staging included 2 cases (18%) of severe dysplasia (CIS), 4 of T1 (36%) and 5 of T2 (45%). The preoperative FOIS and DOSS were both 6.9 and 6.8 out of 7, respectively. All patients underwent intraoperative frozen section analysis. One patient had positive margins that required re‐resection, of which negative margins were obtained. Six patients underwent ipsilateral, and 2 patients underwent bilateral neck dissection at the time of tumor resection. Nine patients additionally underwent sentinel lymph node biopsy. The mean defect size after resection was 4.7 cm (range: 3‐6 cm) length by 3.4 cm (range: 2‐5 cm) width by 0.46 cm (range: 0.1‐0.9 cm) deep, with an average volume defect of 18.4 cm^3^. The maximum tumor depth was 1.15 cm (range: 0.8‐2.0). Postoperative FOIS and DOSS were 4.8 and 5 out of 7, respectively. The patients' hospital stay had a mean length of 1.6 days, with a range of 0 to 3 days. Nine patients (81.8%) required a nasogastric feeding tube postoperatively with a median duration of 8 days (range 7‐30). The 2 patients with CIS did not require the nasogastric tube. Length of follow‐up had a mean of 310 days, with a range of 46 to 798.

**Table 1:** Demographics Information of Patient Cohort

Variable	Total (%)
Mean age	67 years
Sex	
Male	8 (72.7%)
Female	3 (27.2%)
Mean BMI	30.73
Smoking status	
No use	4 (36.4%)
Prior use	5 (45.5%)
Current tobacco use	2 (18.2%)
Chewing tobacco status	
No use	9 (81.8%)
Prior use	1 (9.1%)
Current chewing tobacco use	1 (9.1%)
Alcohol status	
No use	7 (63.6%)
Prior use	3 (27.2%)
Current alcohol use	1 (9.1%)
Mean CCI	3.27
Mean ASA score	2.63

Demographics data and averages of patient cohort.

Abbreviations: ASA, American Society of Anesthesiology Score; BMI, body mass index; CCI, Charlson Comorbidity Index.

Two patients of our cohort (18.2%) experienced site complications, summarized in Table 2. The 2 complications encountered included minor dehiscence in both patients (18.2%) of which 1 patient had self‐resolved bleeding on postoperative Day 11 requiring overnight observation. The second required a readmission for new development of DKA, and incidentally discovery of a seroma in the neck that developed on Day 9. This patient required readmission for 10 days, which led to prolonged nasogastric feeding tube use. No other postoperative complications, such as aspiration, occurred.

**Table 2:** Complications

Complication	Total (%)
Total complications	2 (18.2%)
Dehiscence	1 (9.1%)
Bleeding	1 (9.1%)
Aspiration	0
Seroma	1 (9.1%)
Readmission	1 (9.1%)

Complications experienced by patient cohort

We conducted the telephone encounter, ranging from 2 months to over 12 months postoperatively, and results from this can be found in Table 3. Tongue biting was reported in 3 (27.3%) patients. Dysarthria, secondary to limited tongue mobility, was reported in 3 (27.3%) patients, and was characterized as mild. Food impaction was reported in 2 patients (18.2%). No patient reported any of the issues addressed, including food impaction, during the telephone encounter as a significant problem to their quality of life requiring further workup. FOIS and DOSS mean scores during this encounter was 6.7 and 6.5 respectively (range of 6‐7 FOIS, 4‐7 DOSS).

**Table 3:** Telephone Encounter Summary

Question	Total (%)
Lip biting	
Yes	0
No	11 (100%)
Tongue biting	
Yes	2 (18.2%)
No	9 (81.8%)
Cheek biting	
Yes	1 (9.1%)
No	10 (90.9%)
Food impaction	
Yes	2 (18.2%)
No	9 (81.8%)
External deformity	
Yes	2 (18.2%)
No	9 (81.8%)
Dysarthria	
Yes	3 (27.3%)
No	8 (72.7%)
FOIS	6.7
DOSS	6.5

Telephone encounter summary acquired post‐operatively.

Abbreviations: DOSS, dysphagia outcome severity scale; FOIS, functional oral intake scale.

## Discussion

Priorities for reconstruction after oral tongue defects include adequate elevation and protrusion of the tongue, ensuring proper mobility, and adequate volume restoration of the tongue. These considerations should allow the tongue to be able to contact the palate, which is crucial for adequate food bolus propulsion during swallowing, and functionality in speech and articulation.

Our study determined that the OTAR technique maintains these priorities by preserving swallowing and speech functionality in patients with lateral wedge defects soon after cancer excision. Previous studies have aimed to identify which reconstructive technique yields the most optimal closure and postoperative functional outcomes in patients with cancer‐related tongue excisions. In a review of 43 patients with lateral tongue squamous cell carcinoma, Ji et al divided patients into partial, hemi‐, subtotal, and total glossectomy. They determined that a larger percentage of tongue removed during initial surgery correlated with worsening functional outcomes even 2 to 3 years postoperatively.^4^ Furthermore, a study of 53 patients at 6 months postoperatively demonstrated similar results through that an increased percentage of tissue removed was associated with worse functional outcomes, and techniques that preserved mobility led to improved speech postoperatively,^5^ emphasizing the importance of prioritizing these outcomes through surgeons choice of technique, as demonstrated through the OTAR technique.

Primary closure is a reliable option for partial glossectomy defects, though this technique may often cause limited mobility and deviation of the tongue towards the alveolus with an obliterated lateral sulcus. Primary repair may also lead to tip deviation after large wedge excisions, further impairing future tongue mobility. Adaptations of primary closure techniques, utilizing tongue advancement or tissue movements have been utilized in the past to overcome mobility concerns with primary closure. Such examples include the V to Y closure after tongue base release and rotation, tongue midline split, and tongue base island advancement flaps.^6^, ^7^, ^8^

OTAR can serve as a reliable alternative to simple primary closure or even more complex microvascular techniques for lateral tongue defects after early‐stage (T1/T2) cancer or premalignant lesion resections. For those with later‐stage, larger cancers would still require more complex excision and potential flap reconstruction; however, OTAR is also a relatively straightforward technique with a low learning curve that not only provides adequate defect closure with low perioperative morbidity but also preserves functional swallowing and speech outcomes. These patients' health‐related outcomes most probably are due to the lateral sulcus‐sparing features contributing to tongue mobility as this prevents excessive tugging on the floor of the mouth.

Similar studies support the idea that primary closure techniques may lead to better swallowing post‐operatively for early‐stage tongue cancers. In a study of 49 patients, Ravindra et al divided patients with small to moderate size tongue defects into 3 categories including primary, secondary, and flap reconstruction. They assessed patients with the Speech Intelligibility Assessment score and MD Anderson Dysphagia Inventory at 1 and 6 months postoperatively. They demonstrated that the main advantage of primary closure was that patients had better swallowing postoperatively, compared to both secondary and flap reconstruction. Additionally, patients who did not receive adjuvant treatment had better speech and swallowing scores in the short and long‐term postoperative period, and those without tip of tongue tethering had the best speech and swallowing outcomes.^9^ Another study, by Ochoa et al, with 39 patients who underwent primary closure following resection of early‐stage tongue cancer, also demonstrated patients could expect long‐term swallowing, chewing, and pain to reach near normal at 6 months postoperatively and found there was no significant difference between these parameters in this study population compared to the normative scores published.^10^

In our cohort, we showed site complications such as minor dehiscence in 2 patients (18.2%) of which 1 patient had self‐resolved moderate bleeding on postoperative Day 11 requiring overnight observation. The second requiring a readmission for an unrelated medical issue (diabetic ketoacidosis) and incidental discovery of a seroma. This developed on postoperative Day 9 and the patient required readmission for 10 days. This also led to prolonged nasogastric feeding tube use for a total of 30 days due to intractable vomiting secondary to DKA. No other postoperative complications, such as aspiration, occurred (Table 3).

In this pilot study, we aimed to show the feasibility the OTAR technique in our cohort. The main limitations of this study are the small sample size and the retrospective nature of this study. Another limitation of this study is that our telephone survey was not standardized as we did not conduct them at the same timeline for all patients. The earliest phone call occurred at 2 months postoperatively, whereas the longest phone call was conducted at 12 months postoperatively. In future studies, we hope to address this discrepancy and perform follow‐up questionaries in a standardized timed fashion. Final outcomes generally would be expected to occur at least 1 year after surgery. We chose to limit standardized questions outside of the DOSS and FOIS as our primary aim was on swallowing, and decided to screen for dysarthria using simple subjective questions as this was a pilot study to showcase feasibility of our preferred technique. Future studies could compare OTAR with other techniques of primary closure regarding postoperative quality of life measures.

## Conclusion

OTAR may serve as an effective reconstructive technique for lateral tongue defects suitable for primary closure, preserving functional swallowing and speech outcomes in patients.

## Author Contributions

**Kaersti L. Rickels**, design, conduct, data collection, data analysis, manuscript drafting; **Aryan Shay**, design, conduct, data collection, data analysis, manuscript drafting; **James R. Gardner**, design, conduct, manuscript editing; **Deanne King**, design, conduct, data analysis, manuscript editing; **Jumin Sunde**, design, conduct, manuscript editing; **Mauricio Moreno**, design, conduct, manuscript editing; **Emre Vural**, participating surgeon, design, conduct, data analysis, manuscript editing,

## Disclosure

### Competing interests

None.

### Funding source

None.

## Data Availability

The data presented in this study are available on request from the corresponding author.

## References

[1] De Vicente JC , de Villalaín L , Torre A , Peña I . Microvascular free tissue transfer for tongue reconstruction after hemiglossectomy: a functional assessment of radial forearm versus anterolateral thigh flap. J Oral Maxillofac Surg. 2008;66(11):2270‐2275. 10.1016/j.joms.2008.01.018 18940491

[2] Thompson JA , Vakharia KT , Hatten KM . Advances in oral tongue reconstruction: a reconstructive paradigm and review of functional outcomes. Curr Opin Otolaryngol Head Neck Surg. 2022;30(5):368‐374. 10.1097/MOO.0000000000000828 36004797

[3] Pipkorn P , Rosenquist K , Zenga J . Functional considerations in oral cavity reconstruction. Curr Opin Otolaryngol Head Neck Surg. 2018;26(5):326‐333. 10.1097/MOO.0000000000000474 30024418

[4] Ji YB , Cho YH , Song CM , et al. Long‐term functional outcomes after resection of tongue cancer: determining the optimal reconstruction method. Eur Arch Otrhinolaryngol. 2017;274(10):3751‐3756. 10.1007/s00405-017-4683-8 28748261

[5] Bulbul MG , Wu M , Lin D , et al. Prediction of speech, swallowing, and quality of life in oral cavity cancer patients: a pilot study. Laryngoscope. 2021;131(11):2497‐2504. 10.1002/lary.29573 33881173

[6] Lu J , Chen Y , Xia RH , Shen Y , Zheng Z , Sun J . Modification of the anterior‐posterior tongue rotation flap for oral tongue reconstruction. Head Neck. 2020;42(12):3769‐3775. 10.1002/hed.26409 32767540

[7] Adachi M , Motohashi M , Muramatsu Y . Technique of sliding tongue flap after partial glossectomy. Br J Oral Maxillofac Surg. 2015;53(2):206‐207. 10.1016/j.bjoms.2014.11.006 25480011

[8] Ye W , Hu J , Zhu H , Zhang Z . Tongue reconstruction with tongue base island advancement flap. J Craniofac Surg. 2013;24(3):996‐998. 10.1097/SCS.0b013e31828f1a6b 23714931

[9] Ravindra A , Nayak DR , Devaraja K , Matthew NM , Tiwari S . Functional outcomes after surgical resection of tongue cancer; a comparative study between primary closure, secondary intention healing and flap reconstruction. Indian J Otolaryngol Head Neck Surg. 2022;74(Suppl 3):6296‐6306. 10.1007/s12070-021-03038-1 36742906 PMC9895170

[10] Ochoa E , Larson AR , Han M , et al. Patient‐reported quality of life after resection with primary closure for oral tongue carcinoma. Laryngoscope. 2021;131(2):312‐318. 10.1002/lary.28723 32379355

